# Long-term outcomes after critical illness: recent insights

**DOI:** 10.1186/s13054-021-03535-3

**Published:** 2021-03-17

**Authors:** Anne-Françoise Rousseau, Hallie C. Prescott, Stephen J. Brett, Björn Weiss, Elie Azoulay, Jacques Creteur, Nicola Latronico, Catherine L. Hough, Steffen Weber-Carstens, Jean-Louis Vincent, Jean-Charles Preiser

**Affiliations:** 1 Department of Intensive Care and Burn Center, University Hospital, University of Liège, Liège, Belgium; 2 Department of Internal Medicine, University of Michigan, Ann Arbor, MI USA; 3 Department of Critical Care, Imperial College Healthcare NHS Trust, London, UK; 4 Department of Surgery and Cancer, Imperial College London, London, UK; 5 Department of Anesthesiology and Operative Intensive Care Medicine (CCM, CVK), Charité – Universitätsmedizin Berlin, Berlin, Germany; 6 Freie Universität Berlin, Humboldt-Universität Zu Berlin and Berlin Institute of Health, Berlin, Germany; 7 Réanimation Médicale, Hôpital St Louis, Paris, France; 8 Department of Intensive Care, Erasme University Hospital, Université Libre de Bruxelles, Brussels, Belgium; 9 Department of Anesthesiology, Critical Care and Emergency, Spedali Civili University Hospital, Brescia, Italy; 10 Department of Surgical Specialties, Radiological Sciences and Public Health, University of Brescia, Brescia, Italy; 11 Division of Pulmonary and Critical Care Medicine, Oregon Health and Science University, Portland, OR USA; 12 Erasme University Hospital, Route de Lennik 808, Brussels, Belgium

**Keywords:** Intensive care unit, Post-intensive care syndrome, Critically ill, Core set, Quality of care, ICU-acquired weakness, Muscle weakness, Post-traumatic stress disorder, Follow-up

## Abstract

Intensive care survivors often experience post-intensive care sequelae, which are frequently gathered together under the term “post-intensive care syndrome” (PICS). The consequences of PICS on quality of life, health-related costs and hospital readmissions are real public health problems. In the present Viewpoint, we summarize current knowledge and gaps in our understanding of PICS and approaches to management.

**Supplementary Information:**

The online version contains supplementary material available at 10.1186/s13054-021-03535-3.

## Introduction

The long-term health status of intensive care unit (ICU) survivors has become an increasing concern in recent years, particularly as the number of ICU survivors is increasing as a result of the growing demand for critical care and decreased ICU mortality rates. Regardless of the primary reason for ICU admission, survivors of a prolonged ICU stay may experience medium- and long-term morbidities related to the critical illness, the treatment and organ support received, and the unique ICU environment. The spectrum of morbidities is thus thought to be broader after an ICU stay than after a hospital stay not requiring intensive care [1].

The aim of the present viewpoint is to summarize current knowledge and gaps in knowledge regarding the post-intensive care syndrome (PICS) and potential management strategies.

## Key features of PICS

PICS is a general term referring to new or worsening physical (neuromuscular weakness and reduced autonomy for activities of daily living), mental (anxiety, depression, post-traumatic stress disorder [PTSD]) and neurocognitive disorders that negatively affect daily functioning and quality of life in survivors of critical illness [2]. ICU survivors have a higher risk of death in the years following discharge and a poorer quality of life compared to matched controls [3]. Moreover, PICS can have substantial financial consequences for patients and their families, and there are also considerable economic implications for society as a whole, in terms of, among others, increased healthcare utilization [4].

### Muscle weakness

Persistent ICU-acquired weakness (ICU-AW) is underpinned by a loss of both muscle mass and function, due to combined myopathy and polyneuropathy. Inflammation is a major risk factor for early development of the physiologic alterations that are involved [5]. The neural component includes a channelopathy, implying impaired nerve conduction and primary distal axonal degeneration of motor and sensory fibers, resulting in denervation atrophy [6]. The muscular component typically comprises derangements in excitation–contraction coupling (decreased membrane excitability, injured sarcolemmal membrane, altered calcium homeostasis, disrupted contractile protein interactions) and abnormalities in mitochondrial functional capacities [6]. Neuropathy has been described as inducing more persistent disability than myopathy [7]. Long-term weakness seems to be due also to impaired regenerative capacity: muscle progenitor satellite cell content is decreased in the muscle of ICU survivors, thus compromising muscle regrowth [8].

### Mental disorders

In addition to anxiety [9] and depression [10], psychological stress symptoms are common [11]. Symptoms resolving after a few weeks belong to the “acute stress disorder,” while those persisting for more than 1 month belong to “PTSD-related symptoms.” PTSD symptoms usually appear within three months of the traumatic event, but may occur after a long latent period. Regardless of the duration, re-occurrence of symptoms, avoidance symptoms and constant hyper-arousal symptoms have been reported [12]. Evidence suggests that PTSD is closely related to lack of recollection of the ICU stay: patients with lack of recall or limited factual memories or with delusional or frightening memories are at higher risk for PTSD [13]. Importantly, PTSD-related avoidance symptoms may prevent patients from attending follow-up, thus reducing the possibility of accurate diagnosis and appropriate management.

### Cognitive disorders

Neurocognitive disorders include impaired memory, executive function and mental processing, which can be reported even after a short ICU stay [14]. The risk factors for these impairments are numerous and include hypoxemia, hypotension, glucose dysregulation, drug toxicity (especially sedatives and analgesics) and acute secondary encephalopathies (e.g., delirium) [15]. Systemic inflammation may play an important role, activating brain parenchymal cells with expression of pro-inflammatory mediators within the central nervous system inducing neurotoxicity, endothelial injury and blood–brain barrier dysfunction [15].

## Expanding the definition of PICS

The current, syndrome-based definition of PICS has primarily helped to raise awareness and engagement among all stakeholders (physicians, nurses and (para)medical specialties involved in the recovery process, patients, families, health authorities) [2]. However, PICS may be viewed as an “umbrella term” rather than an actual syndrome, because the underlying mechanisms for the many different components may vary. Indeed, there is huge heterogeneity among survivors of critical illness in terms of expressed components, intensity and duration of “PICS.”

Over the last few years, several new conditions have been described in ICU survivors and have been suggested as additional components of PICS, for example, accelerated bone loss and increased risk of fragility fracture [16], swallowing disorders [17], endocrine and metabolic disorders (including new-onset diabetes [18] and transient alteration of cortisol and anterior pituitary hormones [19]) and sleep disorders [20]. Other post-intensive care sequelae include residual fatigue, defined as a feeling of extreme physical or mental tiredness. It may be explained by axonal losses, but is highly influenced by perceived social support [21]. Persistent pain is also a frequent problem in ICU survivors and can be both nociceptive and neuropathic. Some potential sources of prolonged or chronic pain include poorly treated acute pain with overuse of opioids [22], immobilization and subsequent stiffness of tendons or ligaments, and nerve injuries or degeneration [23]. Importantly, there is a strong interplay between physical, mental and psychological disorders, which merits more attention.

**Fig. 1:**
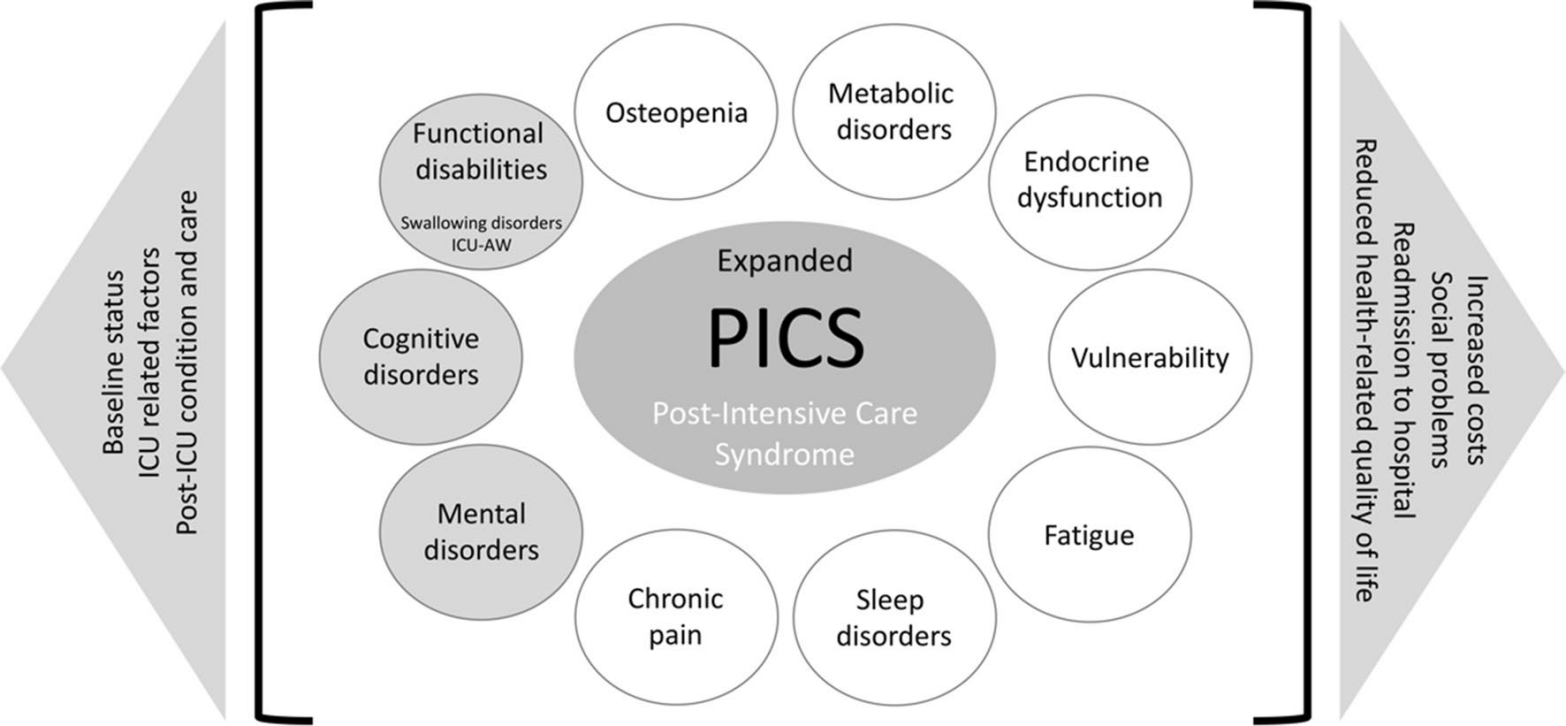
A proposed expanded definition of the post-intensive care syndrome (PICS), including contributing factors (on the left side of the figure) and consequences (on the right side of the figure), current (gray circles) and potential new (white circles) components. ICU-AW; intensive care unit acquired weakness

In view of the growing awareness of these additional post-ICU conditions, it may be time to revise the current syndromic definition. We suggest expanding the definition of PICS to include other domains (Fig. 1) that may be relevant to post-ICU survivors and could further increase awareness of the long-term sequelae of ICU survival. Nevertheless, the validity of this approach needs to be assessed before it can be widely adopted.

## The meaning of “long term”

The meaning of “long term” in relation to PICS is difficult to define and depends on several factors. First, the definition of long term will depend in part on the pre-ICU status. Second, the health trajectory and recovery after ICU admission vary greatly among patients, but there are still no validated tools to estimate potential for recovery. Nevertheless, it is anticipated that previously frail or disabled patients have a reduced chance of recovery compared to previously healthy patients. Advanced age is not always a reliable predictor of future impairment [24]. Third, the time frame also depends on the outcome under consideration: some outcomes can take time to manifest, others occur early but improve rapidly, and others are embedded in multiple domains with different timespans.

## Outcomes assessment

For a given PICS domain, different outcomes can be measured, and, for any given outcome, there are often different ways of measuring it, which may influence the reported incidence of any PICS component. The multitude of possible outcomes and measurement methods thus impacts clinical research, affecting the reproducibility and interpretation of results, and the ability to make comparisons across studies. Moreover, some important outcomes may be unmeasured or unreported. Indeed, post-ICU outcomes measurement remains an important knowledge gap. Creating a core outcome set for components included in an expanded PICS definition could offer a harmonized approach to assessing key dysfunctions and comparing them across studies, and could provide a more accurate picture of each patient’s clinical status. Existing frameworks [25] can help in identifying the most relevant outcomes and could be expanded to include assessment of bone frailty, chronic pain, fatigue, sleep disorders, metabolic and endocrine impairments, and vulnerability (Additional file 1: Table S1). Patients’ opinions as to which outcomes are the most important are crucial when building a core outcome set [26]. Importantly, the most appropriate measurement tools also need to be identified [25]. Measurement tools need to be easy to use, reliable, standardized and able to be repeated at different time points. Ideally, cutoff points for each outcome measure should have been defined prior to inclusion in the core outcome set. Normal ranges can sometimes be deduced from conditions other than post-ICU care. The role of composite measures, measuring multiple aspects of PICS and included in questionnaires or scores, needs to be clarified.

## Mitigating later development of PICS during the ICU stay

The pathophysiology of the sequelae of critical illness that combine to form PICS is complex. Some of the morbidities experienced by ICU survivors after a prolonged stay may be, at least in part, related to metabolic and neuroendocrine derangements perpetuated by unresolved organ failure and inflammation. Although not all contributing factors are modifiable, some clinical phenotypes of PICS may be, at least partly, induced by medical interventions, procedures, medications or even under-treatment during the ICU stay and it may be possible to prevent or at least attenuate some of these factors in individual patients. Some strategies deserve particular attention in the context of mitigating PICS, such as limiting sedation, optimizing nutritional intake, engaging families and promoting exercise.

### Sedation limitation

Deep and prolonged continuous sedation, especially using benzodiazepines, continues to be commonplace in ICU patients, although use of lighter sedation or no sedation has been shown to be safe [27]. Less sedation and benzodiazepine avoidance may be associated with better mental health outcomes [28]. In clinical practice, achieving lighter sedation requires a multimodal approach with adequate pain management, prioritizing patient awakening, educating the ICU team, reassessing the need for sedation on an hourly basis and promoting sleep [29]. Efforts to make the environment and the delivery of care as humane and person-centered as possible within the ICU have positive consequences on the mental health of patients, families and caregivers [30]. Non-pharmacologic interventions, such as hypnosis, music and relaxation techniques, may help reduce pain, agitation and anxiety.

### Nutritional intake

Critical illness is characterized by an uncontrolled catabolic response to stress and anabolic resistance. During the early acute phase following injury, there is endogenous production of glucose that cannot be inhibited by exogenous substrates. Daily energy expenditure increases progressively during the later phases to values higher than before injury [31]. Proteins are lost mainly from skeletal muscle where protein synthesis is also reduced. Optimal nutritional intake aims to support energy and protein requirements to protect against catabolism and to prevent severe deconditioning, while avoiding over-nutrition. Micronutrients are also key components of nutritional support. A high prevalence of vitamin D deficiency has been reported in critically ill patients and there is preliminary evidence that the pleiotropic effects of vitamin supplementation could be beneficial on muscle strength in these patients [32].

### Promoting exercise

To reduce the incidence and severity of muscle wasting and weakness, goal-oriented nutrition is conceptually not sufficient and should be associated with early muscle activation and physiotherapy [33]. Active mobilization and physical rehabilitation in the ICU is possible, safe, and has beneficial effects on short-term ICU outcomes, such as muscle strength at discharge and functional independency [34]. Early mobilization may also provide a physiologic mechanism of overcoming insulin resistance [35]. However, mobilization of ICU patients is not easy. ICU-related processes, structures and culture are the most often reported barriers and careful analysis of these factors should help in implementing early rehabilitation. Issues including which patients, which interventions, and which co-interventions are most likely to be of benefit and when they should be administered need further elucidation. For example, muscle activating measures may augment the positive effects of physiotherapy on myocyte cross-sectional area [36]. Electrical muscle stimulation (EMS) exerts beneficial metabolic effects in muscle cells [37, 38]. However, illness severity seems to influence the contractile response to EMS and may modulate its benefits [39]. Novel methods, such as whole-body vibration, are emerging as possible alternatives for muscle activation [40]; this approach may be particularly suitable for unconscious patients or patients with dressings or wounds that preclude positioning of EMS electrodes.

### Family engagement

The presence of families in the ICU is now encouraged by increasing numbers of guidelines and expert opinion. Unrestricted or flexible visiting hours have the potential to reduce delirium and anxiety symptoms among ICU patients [41]. Families, in conjunction with the caregivers, can also complete ICU diaries, which serve as a record for each patient of all the events occurring during the ICU stay (visits, health status, procedures) and may facilitate psychological recovery after critical illness by helping patients fill gaps in their memories. There are conflicting data on the efficacy of such diaries to prevent or reduce psychological symptoms or PTSD in ICU survivors, which may be related, in part, to different psychological profiles among the studied populations.

## Do patients need follow-up?

ICU survivors are complex patients and, after hospital discharge, may become “lost” in the healthcare system with delays in accessing clinical care that recognizes and proactively addresses their unique limitations and needs. Indeed, a recent cohort study suggests that while recommended practices for post-sepsis care (medication optimization, screening for new impairments, monitoring for common and preventable causes of health deterioration) are associated with improved outcomes, only a minority of patients receive all items [42]. Based on these arguments, a dedicated, multidisciplinary post-ICU follow-up service or clinic to assess and manage PICS problems may be justified. Such services are increasingly available and have been shown to have promising results on mental outcomes [43], although published data in this field are still limited and sometimes controversial, with two randomized controlled trials showing no benefit on selected outcomes of the mode of follow-up tested [44, 45]. The practical modalities of post-ICU clinics are loosely defined and vary widely in terms of involved professionals, patient eligibility, timing and duration of follow-up, and criteria for specialist referral. There are no clear criteria to guide which survivors should be included in follow-up programs. Patients with anticipated better outcomes because of a lower severity of illness or a shorter length of stay should not be overlooked. Intensivists, particularly those with a special interest in this area, may be best qualified to understand all the aspects of critical illness a patient may have encountered and likely post-ICU sequelae, and should probably be key players in the design and running of such services along with nurses and allied health professionals (e.g., pharmacists, physiotherapists, occupational therapists, psychologists). Many primary care physicians or referring specialists are unaware that persistent sequelae may relate to critical illness, and have little time to correctly manage the multiple, often complex, aspects of PICS. Close collaboration between intensivists and other doctors or services involved in home care and social welfare [46] is therefore essential. Indeed, there is a general consensus that the transition from in-hospital to outpatient care could be significantly improved [47], especially in terms of information sharing [48].

Telemedicine is another option for survivor follow-up. Text messaging or smart-phone applications have become widely used interfaces between patients and their providers. High levels of patient satisfaction, time- and cost-saving, and improved access to care are among the numerous benefits of telemedicine. Mindfulness and coping skills training programs delivered by mobile applications have shown encouraging results in reducing psychological distress in survivors and their families [49]. In-home telerehabilitation programs, using telephone and video-based interventions aimed at remediating cognitive, physical and functional deficits, may also be feasible. Telemedicine is also increasingly used for peer support chat within support groups of ICU survivors.

In addition to the diagnosis and management of PICS, post-ICU care should aim to prevent readmissions [50]. Survivors who are particularly frail should be identified early. Medications should be reassessed frequently as vital parameters can be labile in the weeks following hospital discharge, necessitating changes in drug and/or dose. Intervention of an in-hospital clinical pharmacist can help reduce short- and long-term readmissions, especially in patients receiving multiple medications. Vulnerable patients should be educated on risk of re-infection and the swallowing function should be assessed. Physicians should ensure vaccines are up to date, and splenectomized patients are aware of their increased susceptibility to sepsis.

## Conclusions

PICS can be a heavy burden for ICU survivors, their families and society as a whole. There is a broad spectrum of clinical phenotypes, not all of which are correlated to ICU illness severity. Greater awareness of PICS and longer follow-up of ICU survivors has resulted in identification of post-ICU sequelae not included in the current definition, raising the possibility that it should be expanded to include some of these conditions. Development of a core outcome set with appropriate measurement tools would help customize research in this area enabling comparison across studies. Further research is needed to assess the effects of strategies conducted during the ICU stay to prevent or limit the later development of PICS in individual patients. Post-ICU care is still in development with multidisciplinary follow-up likely to be beneficial for patients and families; however, questions remain regarding which patients are most likely to benefit, which outcome definitions and measurements should be used, and which interventions are most likely to be effective.

## Supplementary Information

**Additional file 1**.** Table S1**. Suggested core outcome set in relation to the expanded definition of PICS, and non-exhaustive list of measurement tools that are commonly used in the published studies, with their normal value or range if available.

## Data Availability

Not applicable.

## Abbreviations

EMS: Electrical muscle stimulation
ICU: Intensive care unit
LOS: Length of stay
PICS: Post-intensive care syndrome
PTSD: Post-traumatic stress disorder
