# Supplementary material for: Long-term outcomes after critical illness: recent insights
Source: Crit Care. 2021 Mar 17;25:108. doi: 10.1186/s13054-021-03535-3 (PMC7968190; doi:10.1186/s13054-021-03535-3)
Supplement: Supplementary file 1 — Additional file 1. Table S1. Suggested core outcome set in relation to the expanded definition of PICS, and non-exhaustive list of measurement tools that are commonly used in the published studies, with their normal value or range if available. [file 13054_2021_3535_MOESM1_ESM.pdf]

Long-term outcomes after critical illness: recent insights

Rousseau AF et al

Online Supplementary material

Table S1. Suggested core outcome set in relation to the expanded definition of PICS, and non-exhaustive list of measurement tools that are commonly used in the published studies, with their normal value or range if available.

**Post-ICU outcomes**		**Common measurement tools**	**Normal values where available**
Physical disabilities	Muscle mass	Quadriceps ultrasonography, computerized tomography at lumbar level	
	Muscle strength	Medical Research Council (MRC) Manual Muscle Test<br>Hand grip strength<br>Quadriceps strength	>48<br>> 11 kg in males, > 7 kg in females
	Muscle function	2- or 6-min walking test (2MWT or 6MWT)<br>Time up and go test (TUG)<br>Physical Function in Intensive Care Test scored (PFIT-s)<br>Chelsea Critical Care Physical Assessment tool (CPAx)<br>Short Physical Performance Battery (SPPB)<br>Physical activity questionnaires	depends on age and sex<br>< 13.5 sec, depending on age
	Respiratory function	Modified Medical Research Council Dyspnea Scale<br>Dyspnea-12 questionnaire<br>Diaphragm ultrasonography<br>Maximal inspiratory and expiratory pressures<br>Pulmonary function tests	≥ 10<br><br><br>depends on age, sex, anthropometrics
	Autonomy	Barthel Index<br>Instrumental Activities of Daily Living (IADL)<br>Functional Independence Measure (FIM)	

	Swallowing disorders	Eating Assessment Tool (EAT-10)<br>Water swallow test<br>Bedside swallow evaluation<br>Oropharyngo-esophageal scintigraphy or flexible endoscopic evaluation	< 3
Osteopenia		Bone mineral density (dual-energy X-ray absorptiometry)<br>Bone turnover biomarkers	
Metabolic disorders		Biomarkers<br>Body weight	
Endocrine dysfunction		Biomarkers	
Vulnerability		Clinical Frailty Scale	
Mental disorders		Hospital Anxiety and Depression Scale (HADS)<br>Impact of Event Scale – revised (IES-R)<br>Generalized Anxiety Disorder scale (GAD)<br>Patient health questionnaires	<7 in each subscale<br><22, 1 month after trauma, <36, 3 months after trauma
Cognitive disorders		Montreal Cognitive Assessment (MoCA)<br>Repeatable Battery for the Assessment of Neuropsychological Status (RBANS)	≥ 26
Fatigue		Numeric rating scale<br>Multidimensional Fatigue Inventory (MFI-20)<br>Functional Assessment Chronic Illness Therapy-Fatigue scale (FACIT-F)	 depends on age and sex
Sleep disorders		Pittsburg Sleep Quality Index (PSQI)<br>Epworth Sleepiness Scale	< 5<br>0-6
Chronic pain		Numeric rating scale<br>Brief Pain Inventory – Short Form (BPI-SF)<br>Pain Self-Efficacy Questionnaire (PSEQ) Leeds assessment of neuropathic symptoms and signs (LANSS)	
Quality of life		The Short Form (36) Health Survey (SF-36)<br>EQ-5D instrument	
